# Low expression of the *CCL5* gene and low serum concentrations of CCL5 in severe invasive group a streptococcal disease

**DOI:** 10.1007/s15010-024-02318-6

**Published:** 2024-06-12

**Authors:** V Kailankangas, S Katayama, K Gröndahl-Yli-Hannuksela, J Vilhonen, MH Tervaniemi, K Rantakokko-Jalava, T Seiskari, E Lönnqvist, J Kere, J Oksi, J Syrjänen, J Vuopio

**Affiliations:** 1 Faculty of Medicine and Health Technology, Tampere University, Tampere, Finland; 2 Department of Internal Medicine, Tampere University Hospital, Tampere, Finland; 3 Folkhälsan Research Center, Helsinki, Finland; 4 Stem Cells and Metabolism Research Program, University of Helsinki, Helsinki, Finland; 5 Department of Biosciences and Nutrition, Karolinska Institutet, Solna, Sweden; 6 Institute of Biomedicine, University of Turku, Turku, Finland; 7 Department of Infectious Diseases, Turku University Hospital, Turku, Finland; 8 Faculty of Medicine, University of Turku, Turku, Finland; 9 Department of Clinical Microbiology, Laboratory Division, Turku University Hospital, Turku, Finland; 10 Department of Clinical Microbiology, Fimlab Laboratories, Tampere, Finland; 11 Finnish Institute for Health and Welfare (THL), Helsinki, Finland

**Keywords:** Streptococcus group A, Sepsis, Transcriptome, Streptococcal infections, Critical care

## Abstract

**Purpose:**

Our objective was to elucidate host dependent factors of disease severity in invasive group A Streptococcal disease (iGAS) using transcriptome profiling of iGAS cases of varying degrees of severity at different timepoints. To our knowledge there are no previous transcriptome studies in iGAS patients.

**Methods:**

We recruited iGAS cases from June 2018 to July 2020. Whole blood samples for transcriptome analysis and serum for biomarker analysis were collected at three timepoints representing the acute (A), the convalescent (B) and the post-infection phase (C). Gene expression was compared against clinical traits and disease course. Serum chemokine ligand 5 (CCL5, an inflammatory cytokine) concentration was also measured.

**Results:**

Forty-five patients were enrolled. After disqualifying degraded or impure RNAs we had 34, 31 and 21 subjects at timepoints A, B, and C, respectively. Low expression of the *CCL5* gene correlated strongly with severity (death or need for intensive care) at timepoint A (AUC = 0.92), supported by low concentrations of CCL5 in sera.

**Conclusions:**

Low gene expression levels and low serum concentration of CCL5 in the early stages of an iGAS infection were associated with a more severe disease course. CCL5 might have potential as a predictor of disease severity.

**Summary:**

Low expression of genes of cytotoxic immunity, especially CCL5, and corresponding low serum concentrations of CCL5 associated with a severe disease course, i.e. death, or need for intensive care, in early phase of invasive group A Streptococcal disease.

**Supplementary Information:**

The online version contains supplementary material available at 10.1007/s15010-024-02318-6.

## Introduction

*Streptococcus pyogenes*, or Group A *Streptococcus* (GAS), is among the top ten infectious causes of mortality [1]. It most commonly causes mild to moderate infections of the throat or the skin [2], but also invasive (iGAS) infections, such as septicemia, necrotizing soft-tissue infection, and streptococcal toxic shock syndrome, which are life threatening even when properly treated [3].

Factors affecting disease severity in iGAS infections remain poorly understood. Genetic factors affecting a person’s immune system are thought to play a role on the risk of developing a severe disease course in iGAS disease. There are animal studies indicating this possibility [4–6], but reports on human subjects are scarce, though some exist [7–9].

Transcriptome analysis can be used to obtain information on gene expression, i.e. transcription, in a given context, in a certain cell line at a certain timepoint [10]. It has been shown to be useful in describing the host immune response in septic patients [11, 12]. A transcriptome analysis of leukocyte RNA has been shown to be able to differentiate between a viral and bacterial infection as different genes are activated in each [13, 14]. To our knowledge there are no previous transcriptomic studies in patients with iGAS.

Weighted gene correlation network analysis (WGCNA) is an algorithm increasingly being used in bioinformatics applications. As different genes involved in the same cellular processes and having similar function have been shown to display similar expression profiles, they can be grouped according to co-regulation. WGCNA can be used to find such clusters, or modules, of genes with highly correlated expression profiles, for relating such modules to one another and to external sample traits [15, 16].

In this prospective, 2-year clinical study covering iGAS cases treated in two university hospitals in Finland, we attempted to compare peripheral blood leukocyte transcriptome results of iGAS patients of varying degrees of severity, and compare them at different timepoints, and with WGCNA moduling discover gene expression patterns possibly associated with a more severe disease course. Serum was also collected in case the WGCNA would suggest possible biomarkers.

## Methods

### Study design and patient enrollment

The study design has been described previously [17]. Briefly, the study was conducted as a prospective observational study at two tertiary care Finnish hospitals: Tampere University Hospital in Pirkanmaa Health District and Turku University Hospital in the Hospital District of Southwest Finland between June 2018 and July 2020. A case was defined as a culture positive finding of GAS from a normally sterile site (blood, CSF, pleural fluid, peritoneal fluid, synovial fluid, deep tissue sample) in a patient over 18 years of age.

Upon recruitment, a throat swab was taken (Eswab, Copan), and serum and whole blood samples for RNA isolation (PAXgene Blood RNA tube, BD) were taken at the next convenient time, usually the next morning (timepoint A). Second blood samples (timepoint B) were taken five to seven days later. Three to four months after recruitment each surviving patient was invited for a follow up visit for a third blood sample representing a recovered state and acting as an individualized control (timepoint C).

All collected samples were first sent to University of Turku for storage and further analysis. Throat swabs were cultured and tested with isothermal amplification testing and the results have been reported previously [17]. PAXgene Blood RNA tubes were frozen according to manufacturer’s instructions prior the RNA extraction. Background data were obtained from the interview and electronic patient records with the patients’ consent.

All data were compiled to a REDCap-database accessible only to the researchers. Data analysis was performed using study subject codes without possibility to reveal personal identification.

### Definitions

Severe disease was defined as requiring intensive care or leading to death. Acute kidney injury was defined according to KDIGO [18]. Hypotension was defined as systolic blood pressure below 90 mmHg at admission. Corticosteroid use was defined as administration of supraphysiologic doses of hydrocortisone or prednisolone to treat septic shock or respiratory compromise according to the electronic patient record. The patients’ underlying characteristics were classified according to the Charlson Comorbidity Index. The index was further divided into four categories, 0 score is 0, scores 1–2 are 1, scores 3–4 are 2 and ≥ 5 is 3 [19]. Obesity was defined as Body Mass Index (BMI) *≥* 30 kg/m^2^.

### RNA sequencing

PAXgene Blood RNA tubes were sent to Biomedicum Functional Genomics Unit at University of Helsinki for the analysis. RNA was extracted from the PAXgene Blood RNA tubes (Preanalytix) according to the manufacturer’s instructions.

For RNAseq library preparation, a modified version of the previously described single-cell tagged reverse transcription (STRT) protocol with unique molecular identifiers (UMIs) was used [20]. Forty ng of blood-derived RNA samples were placed on 48-well plates as two libraries with library bias correction [21] with a reagent mix containing primers that attach to the globin transcripts (GlobinLock), inhibiting their reverse transcription [20]. After the GlobinLock addition, oligo-dT primers, template-switching oligonucleotides, and a 6-bp barcode sequence (for sample identification) were added to each well. The synthesized cDNAs were pooled into one library, fragmented to 200–400 bp (Covaris), 5′ fragments were captured, adapters were added, and the targets were amplified by PCR. The RNA-seq libraries were sequenced with Illumina NextSeq 500 System, High Output (75 cycles).

The sequences were processed by STRTprep [22] for quality check and further analysis; hg19 and RefSeq were used as the reference genome and transcriptome, respectively. RNA samples with an RNA integrity number (RIN) < 6 were excluded from library preparation. All the samples from each individual were included in the same library. The library bias in the expression profiles was corrected computationally [21].

The corrected expression levels were normalized by spike-in controls [22] and varied endogenous protein-coding genes at each timepoint were selected by comparison to the technical variations estimated by the spike-ins [22] (*p* < 0.05, adjusted by Benjamini-Hochberg procedure). The selected genes which were significantly altered in at least one timepoint were classified by WGCNA [15]; the soft threshold was 16.

### Serum CCL5 measurements

CCL5/RANTES concentration was measured from serum samples collected at timepoints A, B and C. Commercial enzyme-linked immunoassay was used for the measurement according to manufacturer’s instructions (Human CCL5/RANTES DuoSet ELISA, DY278, R&D Systems).

### Statistical analyses

The summary expression profiles (eigengene) and the associations with clinical traits were estimated by WGCNA; based on the developers’ recommendations, biweight midcorrelation was used for the associations with maxPOutliers = 0.05, and robustY = FALSE for the association to the binary clinical traits. For the prediction of a clinical trait, the probability was modeled by logistic regression. Gene set enrichment analysis was performed on Enrichr [23] for Gene Ontology biological terms.

Using a logistic regression analysis [24], the expression levels were compared against the binary categories of need for intensive care, death, severe disease (a composite of the prior two), acute kidney injury, hypotension, and the presence of GAS in the throat swab, as well as possible confounding factors such as age, gender, obesity, use of corticosteroids, and underlying conditions as defined by the Charlson comorbidity index. They were also compared against CRP and leukocyte levels at timepoint A.

Unpaired, nonparametric Mann-Whitney U test (GraphPad Prism version 9.0) was used to calculate differences in CCL5 concentrations between patient groups severe and nonsevere disease. A p-value of < 0.05 was considered significant. Pearson’s bivariate correlation (IBM SPSS Statistics) was used to check for a correlation between the CCL5 serum concentration and *CCL5* expression level.

## Results

### Patient enrollment, clinical characteristics, and disease severity

Altogether 45 patients were enrolled. Details of clinical characteristics and infection foci have been described earlier [17]. Twenty-seven patients (60%) were male and 18 (40%) female. The mean and median age was 55 years. The most common underlying conditions were obesity (31%) and hypertension (31%), while 40% of the patients had no previously diagnosed chronic conditions. 20% (9/45) of patients were in Charlson class 0, 42% (19/45) in class 1, 13% (6/45) in class 2 and 24% (11/45) in class 3.

The most common infectious focus was soft tissue infection (51%). Thirteen cases (29%) needed intensive care. Eight patients (18%) died before the follow-up visit: four within a week from hospital admission, and an additional three later during hospitalization. One patient died over a month after the acute phase.

Sufficient PAXgene samples were acquired from 41 cases at timepoint A, 35 at timepoint B and 26 at timepoint C. Serum samples were obtained from 42 patients at timepoint A, 35 at timepoint B and 26 at timepoint C (Fig. 1).

**Figure:**
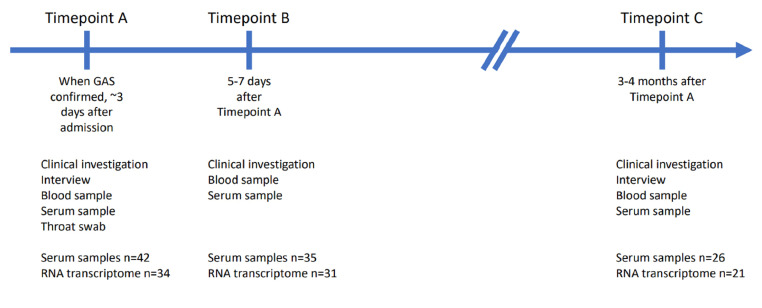
Figure 1

Figure 1 Overview of the study design. When GAS was isolated from blood or other normally sterile site, the patient would be recruited, interviewed, assessed clinically, and a throat swab, serum, and whole blood (PAXgene Blood RNA tube, BD) to be used for transcriptome analysis would be obtained (timepoint A). Five to seven days later a second blood test was taken (timepoint B). Three to four months after recruitment each patient was invited for a follow up visit to obtain a third blood test to represent the patients’ transcriptome in a recovered state (timepoint C). The numbers of RNA transcriptome samples represent the samples that were left after disregarding impure or insufficient samples.

### Transcriptome analysis results

After quality trimming of the RNA samples, 94 were subject to transcriptomic analysis. Eight out of the 94 samples were disqualified for further analysis due to low spike-in reads or low 5’-end capture rates in the protein-coding genes, suggesting assay failure. The remaining 86 qualified samples included 34, 31, and 21 subjects at timepoints A, B and C, respectively.

**Fig. 2:**
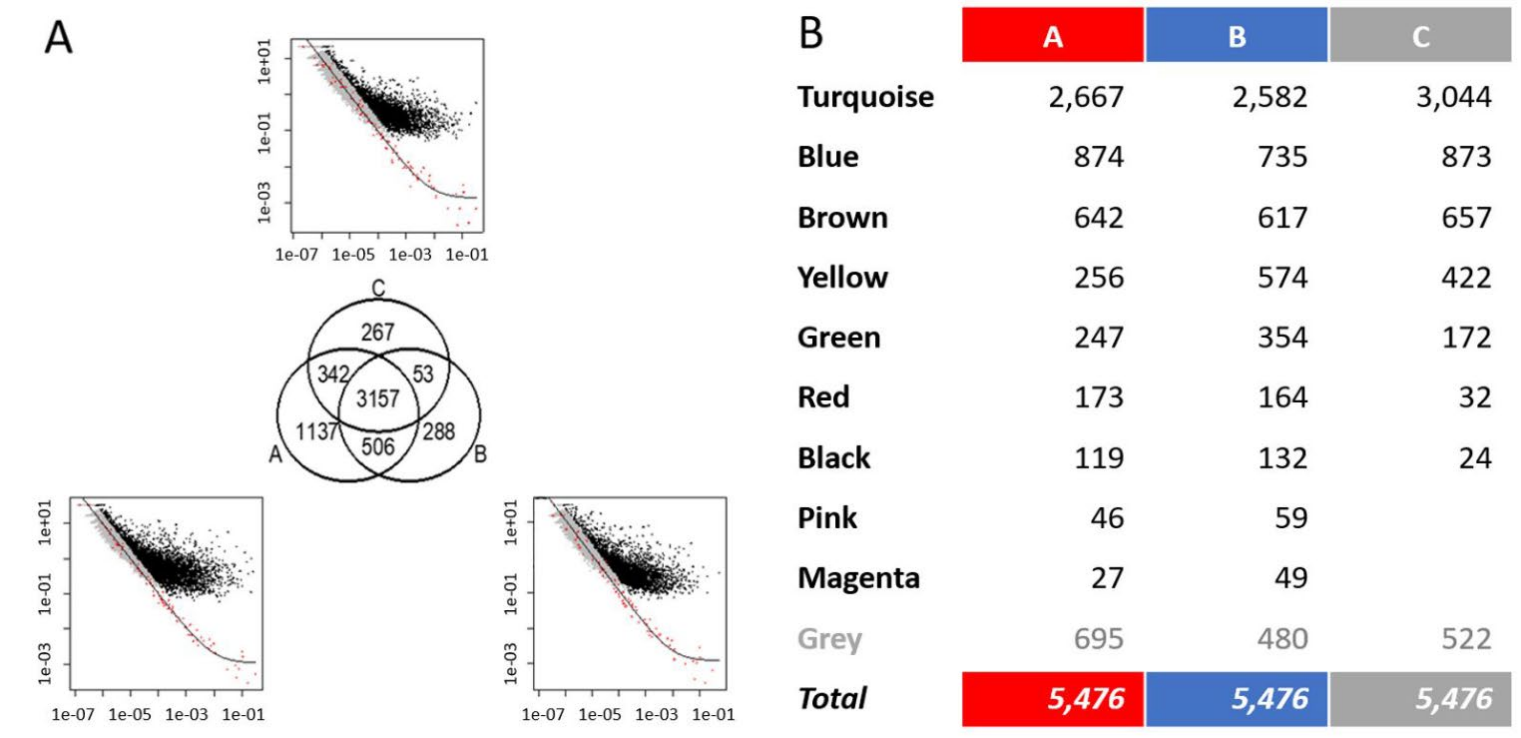
Overview of the transcriptome data. (**A**) 5,476 genes altered in timepoints A, B and C. Each scatter plot represents the significantly altered genes (black) in each timepoint compared to the technical variations of the spike-in RNAs (red). Y axes are the coefficient of variation, X axes are normalized mean expressions. (**B**) Modules (row header, color names) and the number of the selected genes in each group and at each timepoint. Genes in the grey group are unclassifiable

Out of the 15,589 protein-coding genes and spike-in RNAs yielding sequence, 5,476 genes with biological variation in the qualified samples at each timepoint were selected [Fig. 2a] and classified into ten modules by WGCNA according to the expression correlation [Fig. 2b]. The modules were arbitrarily assigned a color code as an identifier. Although the genes selected from the timepoints were similar, the Uniform Manifold Approximation and Projections (UMAP) of these selected genes suggested large timepoint-dependent effects and associations with disease severity (Supplementary Fig. 1).

**Fig. 3:**
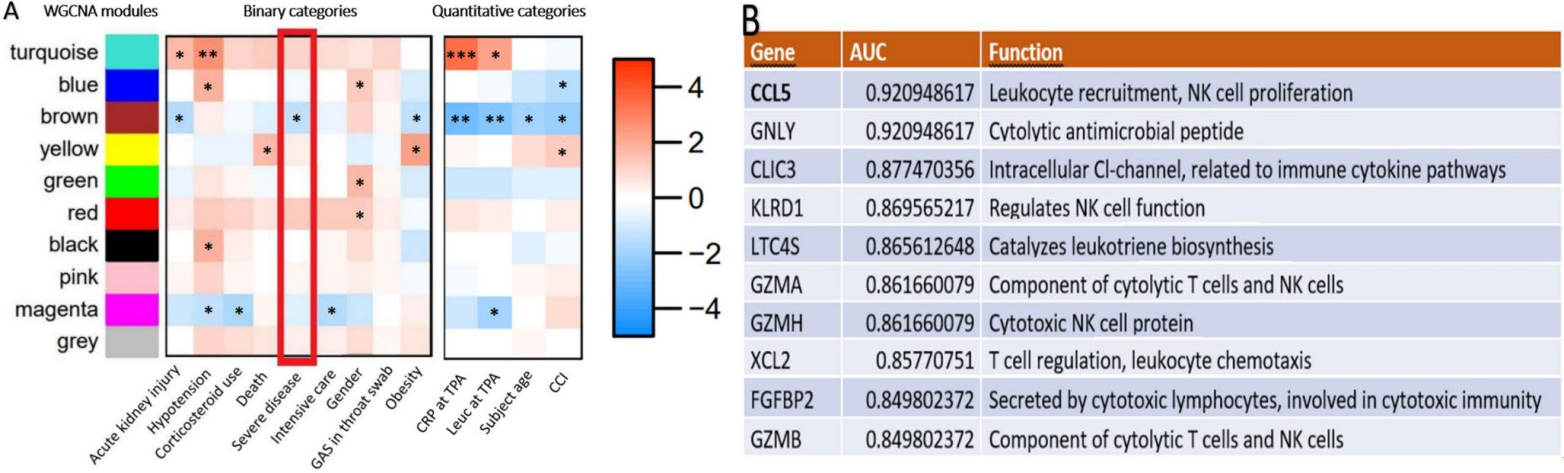
Associations between the color-coded modules and single genes in the brown module. (**A**) Associations of the modules with correlating gene expression levels, and the clinical traits at timepoint A. The severe disease category is highlighted. The color gradient signifies values of -log10(P) * sig(r), where P is P-value of a correlation coefficient between expression levels of a module and clinical parameters, r is the correlation coefficient, and sig(r) is 1 if *r* > 0 (= positive correlation) and − 1 if *r* < 0 (= negative correlation). The exclamation marks signify statistical significance, * = *p* < 0.05, ** = *p* < 0.005, *** = *p* < 0.0005. WGCNA = weighted gene correlation network analysis, Obesity = BMI *≥* 30 kg/m^2^, CRP = C-reactive protein, Leuc = leucocyte count, CCI = Charlson comorbidity index, TPA = timepoint A. (**B**) Top 10 associations of low expression of single genes with severity in the brown module and their functions.

Together with the grouping of the selected genes, a summary expression profile of each timepoint, consisting of the color-coded modules, was estimated and compared with nine binary and four quantitative clinical traits (Fig. 3a). We focused primarily on severe disease, death and need for intensive care at timepoint A, representing the acute phase.

**Fig. 4:**
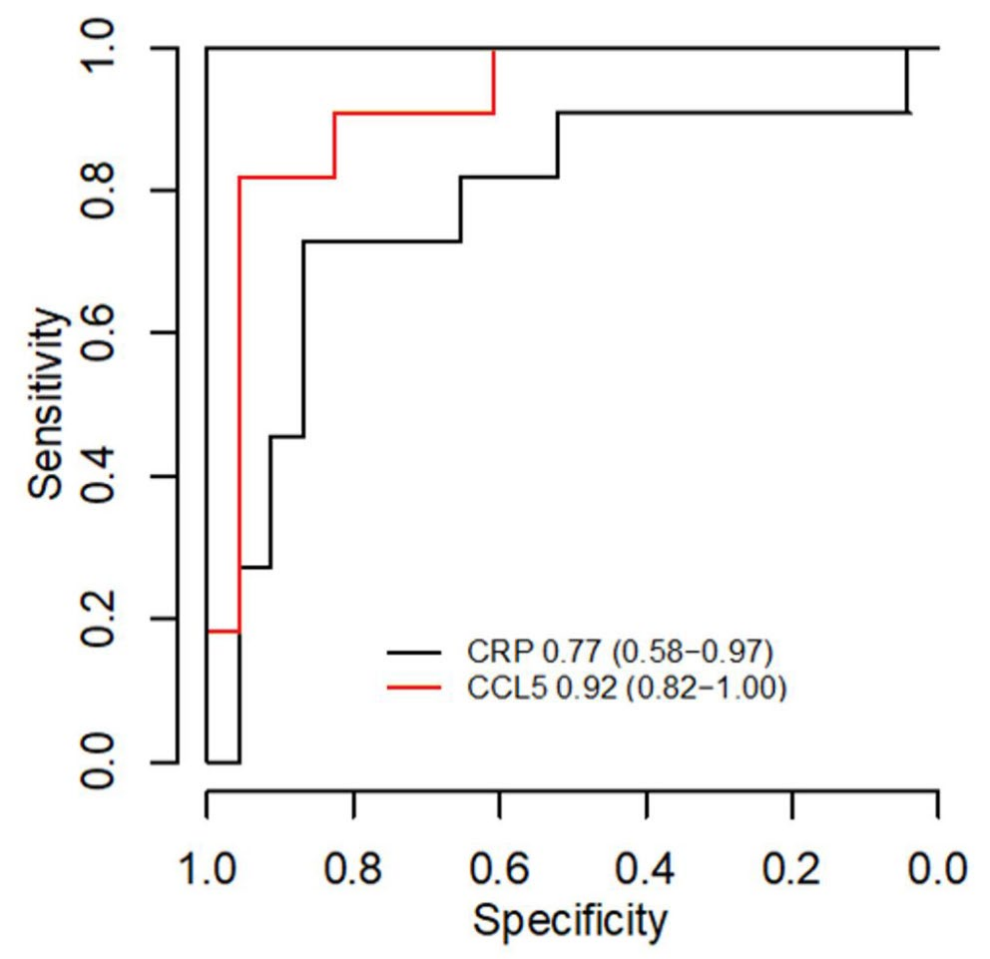
Receiver operating characteristic curves of *CCL5* expression compared to CRP. AUC and the 95% CI are beside the legends. At 90% specificity, the sensitivity of *CCL5* expression for severe disease is 80%, whereas for CRP it is 45%

At timepoint A, severe disease was most strongly associated with low expression levels of genes in the brown module. The same association was found with acute kidney injury. The ten genes with the strongest association with severe disease in the brown module were related to natural killer cell (NK cell) functions or otherwise to cytolytic immunity (Fig. 3b) [25–32]. Of these, low expression level of *CCL5* had the strongest correlation to severe disease (AUC 0.92, 0.82-1.00 in 95% CI; Fig. 4). Low expression levels of the brown module were also associated with a high Charlson comorbidity index, as well as older age, and higher C-reactive protein (CRP) and leukocyte values. The second strongest associations with severe disease were high expression of the genes in the turquoise and red modules (Supplementary Tables 1 and 2). High expression of the turquoise module also associated with acute kidney injury, hypotension and high CRP and leukocyte counts (Fig. 3a). Expression levels of two genes, one from brown and the other one from turquoise or red, at timepoint A had an even stronger association with severe disease (Supplementary Table 3), but these associations were less robust because of the number of events per variable (EPV) was less than 20 as would be ideal for a two gene analysis [24].

The yellow and magenta modules (Fig. 3b; Supplementary Table 4) were associated with death and intensive care, respectively. High expression of the yellow module was strongly linked to death, and low expression of the magenta to need for intensive care, although the EPVs were also less than 10.

None of the top ten genes in the brown module carried the associations over to timepoints B or C, nor did the top ten genes in the turquoise or red module.

### CCL5 measurement

As the transcriptome data showed low *CCL5* expression to be associated with severe disease, the soluble CCL5 concentrations were measured from the serum samples collected at all three timepoints. CCL5 concentration was measured from 103 serum samples from 42 enrolled patients: 42 subjects at timepoint A, 35 at timepoint B and 26 at timepoint C. Twenty-five patients had serum available from all three timepoints, timepoints A + B from 10 subjects, timepoints A + C from one subject and solely timepoint A from six subjects.

**Fig. 5:**
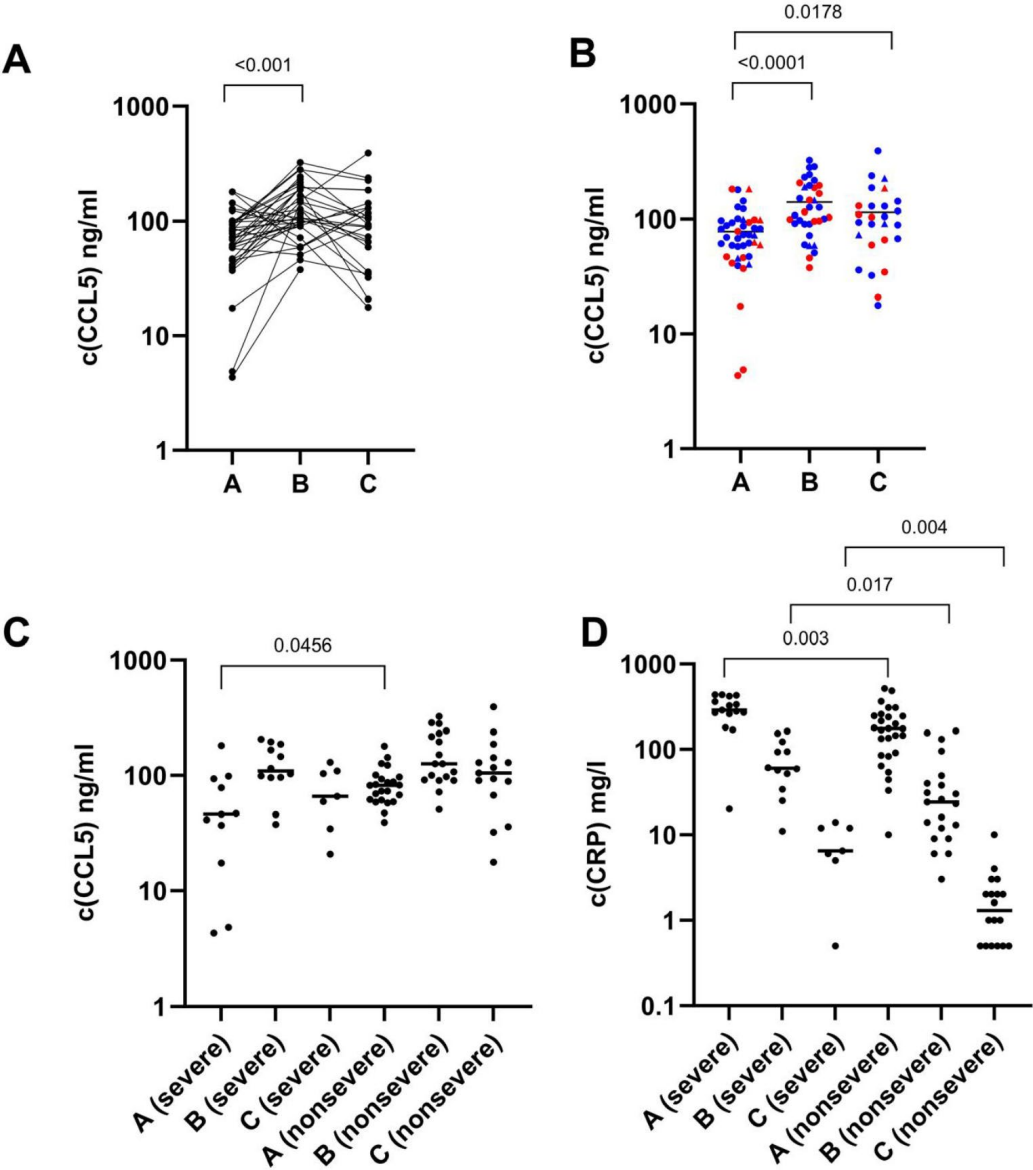
CCL5 concentrations measured from the serum samples of the patients at timepoints A (*n* = 42), B (*n* = 35) and C (*n* = 26). (**A**) Individual cases CCL5 concentration trends. p-values: Paired t-test. (**B**) Dots represent the patients whose samples were included in the RNA transcriptomic analysis, triangles represent patients with missing transcriptome data. Red indicates severe disease. Black line indicates the median. p-values: Mann-Whitney U test. (**C**) Comparison between CCL5 concentration of the severe and nonsevere groups in the three timepoints, included are only cases with available transcriptome data. At timepoint A there is a statistically significant difference in CCL5 concentrations between severe (11 cases) and nonsevere (23 cases) groups (46.2 vs. 81.8 ng/ml, respectively, *p* = 0.0456). Black line indicates the median. p-values: Mann-Whitney U test. (**D**) Comparison of C-reactive protein concentrations between the severe and nonsevere groups in the three timepoints. All cases with available C-reactive protein concentration data shown. Black line indicates the median. p-values: Mann-Whitney U test

Median CCL5 concentration of the whole population increased significantly from timepoint A to timepoint B (median 72.8 vs. 115 ng/ml, respectively, *p* < 0.0001, Fig. 5a and b). CCL5 concentration was still significantly elevated at timepoint C compared with timepoint A (98.6 vs. 72.8 ng/ml, respectively, *p* = 0.0178, Fig. 5b). At timepoint A, when comparing CCL5 concentrations from only cases with available transcriptome data, there was a statistically significant difference between CCL5 levels among the 11 severe and 23 nonsevere cases (46.2 ng/ml vs. 81.8 ng/ml, respectively, *p* = 0.046, Fig. 5c).

The four patients with the lowest serum levels at timepoint A all had severe disease. Of the cases in the lowest 25 percentile (< 47ng/ml) of CCL5 levels with or without transcriptome data at timepoint A, 7/10 were in the severe disease group. The correlation coefficient for the expression level and serum concentration was 0.336 (*p* = 0.052). Median CRP concentrations were also statistically significantly different between the severe and nonsevere groups at each timepoint.

## Discussion

The pathophysiology of severe disease in iGAS infections remains poorly understood. In recent years, metagenomics and machine learning have widened our understanding of host immunologic processes and pathogen related factors. However, to our knowledge studies of transcriptome activity in iGAS patients have not been previously conducted.

CCL5, also known as RANTES (Regulated upon Activation, Normal T-cell Expressed and Secreted), a gene expressed by T lymphocytes, macrophages and other immune active cell types, plays an active role in recruiting leukocytes into inflammatory sites, and inducing proliferation of NK cells [25]. In our study we found a definite correlation between low expression levels of *CCL5* and disease severity in the early stage of the disease. A similar association has been previously described in sepsis [26]. Low *CCL5* expression levels were also associated with a higher Charlson comorbidity index and age, indicating that comorbidities and senescence might impair its expression. CCR5, of which CCL5 is a ligand, has been shown to also serve as a receptor for *S. aureus* cytotoxins [33]. Natural ligands of CCR5, such as CCL5, were shown to reduce this cytotoxicity. CCL5 blockade has also been shown to produce lethal pneumonia in mice with *S. pneumoniae* carriage [34]. This may suggest that GAS toxins might have similar affinity to CCR5, and low levels of the natural ligands thus increase susceptibility to severe disease, or that GAS toxins flooding of CCR5 causes downregulation of CCL5 through a feedback mechanism.

In addition, the difference was also seen in the CCL5 serum concentrations of the severe and nonsevere groups at timepoint A. Thus, low levels of CCL5 in the serum may be suggestive of a severe disease course in the early stage. CCL5 levels were lower in both groups at timepoint A as opposed to timepoints B and C indicating differing kinetics in the first days of iGAS infection. The ICU treated cases who survived until timepoint B were able to catch up to the CCL5 production of the nonsevere cases. This reflects the behavior of the expression levels between timepoints. A statistical correlation between the expression level and serum concentration was not seen, however. CCL5 can be secreted by cells other than the peripheral blood leukocytes represented in the transcriptome analysis. Moreover, we do not know whether the expression levels among our cases are going up or down, and whether the serum concentrations are going up or down at timepoint A. It can be assumed that the serum concentrations would follow the expression levels after a delay depending on CCL5 half-life and other factors. Unlike with the expression level, the serum concentration did not have a stronger association with disease severity than CRP.

All the other genes in the cluster associated with severe disease were also associated with cytotoxic immunity and NK cell function, and most have been previously described as having an inverse relationship with sepsis severity [25–32].

No associations highlighted at timepoint A carried over to timepoint B. This, and the behavior of CCL5 serum levels at timepoint A versus timepoint B, suggest that the mechanism for developing a severe disease course early on may be markedly different from the mechanism underlying a delayed death from the illness, as has been previously suggested [35]. The association between low expression levels of this module with acute kidney injury, and with high levels of CRP, leukocytes is likely reflective of the same association with disease severity.

None of the genes associated with severe disease at timepoint A retained this association among the survivors at timepoint C representing a recovered state. This suggests that the expression levels of the pertinent genes would not have differed at baseline. An alternative explanation could be that the cases with a nonsevere disease had greater expression levels of these genes at timepoint A and then reverted to baseline, whereas the cases with a severe disease were perhaps, for unclear reasons unable to increase their expression of these genes from baseline.

A main limitation of our study is the delay from hospital admission to timepoint A, due to the inability of current diagnostic tools to distinguish iGAS patients from other patients immediately upon admission. Therefore, timepoint A does not represent the onset phase of the disease, but instead on average two to three days after admission. Consequently, we cannot draw a definite conclusion from this data how the transcriptome or CCL5 serum concentration would behave in the very early stages. The lack of healthy controls, especially for the behavior of CCL5 serum concentration, is also limiting. Furthermore, since our study only recruited iGAS patients, we do not know how the concentration would behave with other pathogens. Another limitation is the sample size, limiting power for statistical comparisons. Ideally there would be ten or more cases for a statistically robust logistic regression analysis [24]. For this reason, we used the composite category for severe disease, with eleven cases, to improve reliability of the analysis.

## Conclusions

As previously described in sepsis caused by other pathogens, low expression levels of genes associated with NK cell function and cytotoxic immunity are associated with a severe disease course also among patients with an iGAS disease. We detected low levels of *CCL5* expression and corresponding low CCL5 serum levels. These biomarkers may be possible predictors of a severe disease course in the early stages of iGAS infection. However, we cannot know from our data whether low expression leads to severe disease or is caused by it. This is likely not a GAS specific association, and further studies are warranted to outline the behavior of CCL5 and other associated genes in the very first days of symptoms in iGAS infections as well as other bacteremic infections. It is possible, that low serum levels of CCL5 result from depletion or impaired CCL5 expression. Polymorphism of *CCL5* expression or function as a risk factor for severe iGAS infection could also serve as a future research direction.

## Electronic supplementary material

Below is the link to the electronic supplementary material.

Supplementary Material 1

## Data Availability

Based on the ethical clearance, the datasets generated during the current study are not publicly available as they contain sensitive health related data.
