# Supplementary material for: Low expression of the CCL5 gene and low serum concentrations of CCL5 in severe invasive group a streptococcal disease
Source: Infection. 2024 Jun 12;53(1):51–9. doi: 10.1007/s15010-024-02318-6 (PMC11825563; doi:10.1007/s15010-024-02318-6)
Supplement: Supplementary file 1 — Supplementary Material 1 [file 15010_2024_2318_MOESM1_ESM.docx]

**Supplementary images and tables**


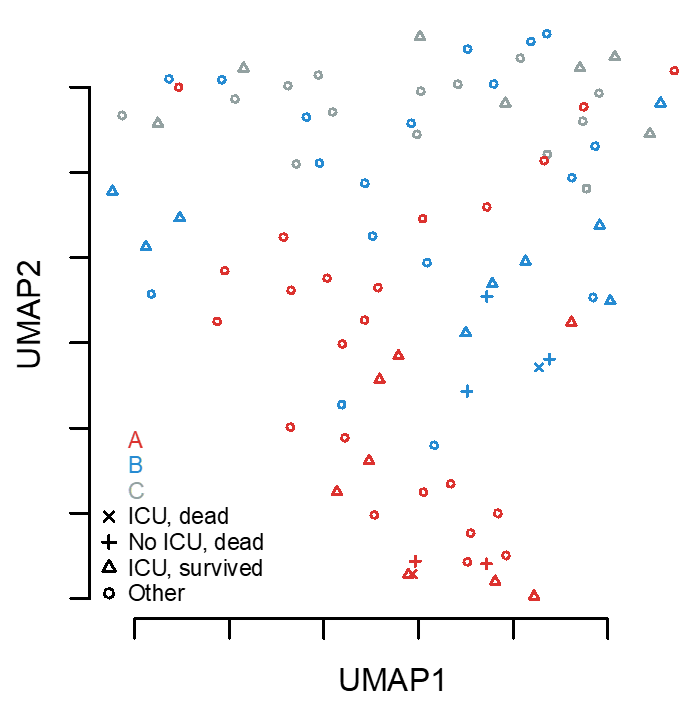


**Supplementary figure 1.** Overview of the qualified samples by uniform manifold approximation and projection on the expression patterns of the selected genes. The colors represent the different timepoints. The samples in the category “Other” are the cases defined as nonsevere, the other samples are in the severe group.

| **Turquoise** | **AUC** | **Red** | **AUC** |
| --- | --- | --- | --- |
| **OLAH** | 0.924901186 | **UBL5** | 0.885375494 |
| ST6GALNAC3 | 0.901185771 | BIRC2 | 0.865612648 |
| NECAB1 | 0.889328063 | UQCR11 | 0.861660079 |
| S100A8 | 0.889328063 | CBX3 | 0.85770751 |
| CNIH4 | 0.877470356 | ATP5F1E | 0.849802372 |
| TXN | 0.877470356 | ATP5MPL | 0.833992095 |
| IL18R1 | 0.873517787 | PET100 | 0.822134387 |
| SPRY1 | 0.873517787 | NDUFB1 | 0.81027668 |
| GAS7 | 0.865612648 | TPT1 | 0.81027668 |
| MTRNR2L2 | 0.865612648 | GTPBP10 | 0.806324111 |

**Supplementary table 1.** Top ten genes associated with severe disease in the turquoise and red modules. AUC = area under the receiver operating characteristic curve.

| **Term** | **Overlap** | **Adjusted p-value** | **Odds Ratio** |
| --- | --- | --- | --- |
| cytoplasmic translation (GO:0002181) | 36/93 | 2.50E-26 | 20.1156853 |
| SRP-dependent cotranslational protein targeting to membrane (GO:0006614) | 35/90 | 6.43E-26 | 20.236783 |
| peptide biosynthetic process (GO:0043043) | 44/162 | 1.21E-25 | 11.9970523 |
| cotranslational protein targeting to membrane (GO:0006613) | 35/94 | 1.91E-25 | 18.8608885 |
| translation (GO:0006412) | 49/214 | 2.90E-25 | 9.61170218 |
| protein targeting to ER (GO:0045047) | 36/103 | 2.93E-25 | 17.1044776 |
| nuclear-transcribed mRNA catabolic process, nonsense-mediated decay (GO:0000184) | 36/113 | 1.01E-23 | 14.8754018 |
| rRNA processing (GO:0006364) | 43/173 | 1.01E-23 | 10.6177475 |
| rRNA metabolic process (GO:0016072) | 41/162 | 6.19E-23 | 10.8457942 |
| ribosome biogenesis (GO:0042254) | 44/192 | 7.87E-23 | 9.55030281 |
| **Term** | **Overlap** | **Adjusted p-value** | **Odds Ratio** |
| neutrophil mediated immunity (GO:0002446) | 250/488 | 1.18E-88 | 7.42943019 |
| neutrophil activation involved in immune response (GO:0002283) | 249/485 | 1.18E-88 | 7.46020839 |
| neutrophil degranulation (GO:0043312) | 247/481 | 4.18E-88 | 7.45824151 |
| cytokine-mediated signaling pathway (GO:0019221) | 191/621 | 1.84E-27 | 3.03234117 |
| ubiquitin-dependent protein catabolic process (GO:0006511) | 114/354 | 2.19E-17 | 3.18024873 |
| antigen processing and presentation of exogenous peptide antigen via MHC class I (GO:0042590) | 44/78 | 2.62E-16 | 8.53486129 |
| protein transport (GO:0015031) | 113/369 | 1.74E-15 | 2.95140741 |
| proteasome-mediated ubiquitin-dependent protein catabolic process (GO:0043161) | 101/321 | 1.26E-14 | 3.06173918 |
| antigen processing and presentation of exogenous peptide antigen via MHC class I, TAP-dependent (GO:0002479) | 40/73 | 2.95E-14 | 7.98237418 |
| endoplasmic reticulum to Golgi vesicle-mediated transport (GO:0006888) | 69/185 | 8.13E-14 | 3.94193478 |
| **Term** | **Overlap** | **Adjusted p-value** | **Odds Ratio** |
| aerobic electron transport chain (GO:0019646) | 16/70 | 4.21E-16 | 37.3163482 |
| mitochondrial ATP synthesis coupled electron transport (GO:0042775) | 16/71 | 4.21E-16 | 36.6360162 |
| cytoplasmic translation (GO:0002181) | 17/93 | 1.02E-15 | 28.3204285 |
| translation (GO:0006412) | 21/214 | 4.18E-14 | 14.0548814 |
| cotranslational protein targeting to membrane (GO:0006613) | 15/94 | 5.17E-13 | 23.7317738 |
| protein targeting to ER (GO:0045047) | 15/103 | 1.77E-12 | 21.2949511 |
| SRP-dependent cotranslational protein targeting to membrane (GO:0006614) | 14/90 | 4.61E-12 | 22.8826548 |
| peptide biosynthetic process (GO:0043043) | 16/162 | 8.37E-11 | 13.7377192 |
| ribosome biogenesis (GO:0042254) | 17/192 | 8.38E-11 | 12.2375092 |
| nuclear-transcribed mRNA catabolic process, nonsense-mediated decay (GO:0000184) | 13/113 | 1.43E-09 | 16.0281875 |

**Supplementary table 2.** Gene ontology biological process terms for brown, turquoise and red module’s genes at timepoint A. Overlap means the overlap rate with member genes of the gene ontology term, the denominator means the number of total genes with which the biological term is associated, and the numerator is the number of genes in the module associated with the term.

| **Gene 1** | **Module** | **Gene 2** | **Module** |
| --- | --- | --- | --- |
| CCL5 | Brown | ABCC5 | Turquoise |
| CCL5 | Brown | ABRACL | Turquoise |
| CCL5 | Brown | AIM2 | Turquoise |
| CCL5 | Brown | ASB3 | Turquoise |
| CCL5 | Brown | ATP5F1E | Red |
| CCL5 | Brown | C18orf32 | Turquoise |
| CCL5 | Brown | C7orf25 | Turquoise |
| CCL5 | Brown | C8orf44 | Turquoise |
| CCL5 | Brown | CCDC32 | Turquoise |
| CCL5 | Brown | CDKN2C | Turquoise |
| CCL5 | Brown | CETN3 | Red |
| CCL5 | Brown | CGRRF1 | Turquoise |
| CCL5 | Brown | CLK1 | Turquoise |
| CCL5 | Brown | COQ10B | Turquoise |
| CCL5 | Brown | COX19 | Turquoise |
| CCL5 | Brown | CRIPT | Red |
| CCL5 | Brown | DCK | Turquoise |
| CCL5 | Brown | DNAJA2 | Turquoise |
| CCL5 | Brown | DNAJB9 | Turquoise |
| CCL5 | Brown | EAF2 | Turquoise |
| CCL5 | Brown | GNG2 | Turquoise |
| CCL5 | Brown | GTF2B | Turquoise |
| CCL5 | Brown | HSPB11 | Red |
| CCL5 | Brown | INO80C | Turquoise |
| CCL5 | Brown | LARP7 | Red |
| CCL5 | Brown | MDP1 | Red |
| CCL5 | Brown | MED16 | Turquoise |
| CCL5 | Brown | MED21 | Turquoise |
| CCL5 | Brown | MFAP1 | Turquoise |
| CCL5 | Brown | MGAT1 | Turquoise |
| CCL5 | Brown | MOB4 | Turquoise |
| CCL5 | Brown | MRPL33 | Turquoise |
| CCL5 | Brown | MRPL50 | Turquoise |
| CCL5 | Brown | PAIP2 | Turquoise |
| CCL5 | Brown | PCMTD1 | Turquoise |
| CCL5 | Brown | PMAIP1 | Turquoise |
| CCL5 | Brown | POLR2K | Red |
| CCL5 | Brown | PSMA3 | Red |
| CCL5 | Brown | PSMD10 | Turquoise |
| CCL5 | Brown | PTP4A2 | Turquoise |
| CCL5 | Brown | RCHY1 | Turquoise |
| CCL5 | Brown | RHEB | Turquoise |
| CCL5 | Brown | RPL21 | Red |
| CCL5 | Brown | RWDD1 | Red |
| CCL5 | Brown | SARAF | Turquoise |
| CCL5 | Brown | SLC25A53 | Turquoise |
| CCL5 | Brown | SLC40A1 | Turquoise |
| CCL5 | Brown | SMIM20 | Turquoise |
| CCL5 | Brown | SRP19 | Red |
| CCL5 | Brown | TBPL1 | Turquoise |
| CCL5 | Brown | TNFAIP6 | Turquoise |
| CCL5 | Brown | TPT1 | Red |
| CCL5 | Brown | UBL5 | Red |
| CCL5 | Brown | UQCR11 | Red |
| CCL5 | Brown | VAMP4 | Turquoise |
| CCL5 | Brown | YBX1 | Turquoise |
| CCL5 | Brown | YIPF5 | Turquoise |
| CCL5 | Brown | ZNF330 | Red |
| CD99 | Brown | DCTN5 | Turquoise |
| CD99 | Brown | DNAJA2 | Turquoise |
| CISD3 | Brown | ATP5F1E | Red |
| CISD3 | Brown | PFDN5 | Red |
| CISD3 | Brown | RBX1 | Red |
| CISD3 | Brown | RPL41 | Red |
| CORO1B | Brown | DNAJA2 | Turquoise |
| CORO1B | Brown | FYTTD1 | Turquoise |
| CORO1B | Brown | TEX30 | Turquoise |
| CORO1B | Brown | VAMP4 | Turquoise |
| DGCR6L | Brown | MGAT1 | Turquoise |
| DNPH1 | Brown | ZNF330 | Red |
| EIF2B3 | Brown | ATP5F1E | Red |
| EIF2B3 | Brown | RCHY1 | Turquoise |
| EXOSC8 | Brown | ATP5F1E | Red |
| FGFBP2 | Brown | VAMP4 | Turquoise |
| GSTP1 | Brown | ZNF330 | Red |
| GZMB | Brown | SRP19 | Red |
| GZMH | Brown | CDKN2C | Turquoise |
| HLA-DPA1 | Brown | ATP5F1E | Red |
| HLA-DPA1 | Brown | SKP1 | Turquoise |
| HLA-DPB1 | Brown | SKP1 | Turquoise |
| PLEKHO1 | Brown | ATP5F1E | Red |
| PLEKHO1 | Brown | RBX1 | Red |
| PLEKHO1 | Brown | UQCR11 | Red |
| PNKD | Brown | SRP19 | Red |
| SGF29 | Brown | GABARAPL2 | Turquoise |

**Supplementary table 3.** Strongest associations of two gene combinations versus severe disease (death or need for intensive care) at timepoint A. All listed pairs of genes produced an area under the receiver operating characteristic curve of 1.00. This would be less robust because of the number of events per variable (EPV) was less than 10 [24].

| **Yellow** | **AUC** | **Magenta** | **AUC** |
| --- | --- | --- | --- |
| KREMEN1 | 0.989247312 | TOX | 0.764444444 |
| ELF3 | 0.989247312 | LBX2 | 0.751111111 |
| BCLAF3 | 0.989247312 | P4HTM | 0.742222222 |
| GALNT15 | 0.989247312 | PCGF6 | 0.737777778 |
| SH3GL3 | 0.967741935 | EPHX4 | 0.706666667 |
| SLC35F | 0.967741935 | FCSK | 0.706666667 |
| SORBS1 | 0.967741935 | CRB3 | 0.688888889 |
| DTWD2 | 0.967741935 | PDCD1 | 0.671111111 |
| TELO2 | 0.967741935 | SERPINC1 | 0.657777778 |
| CFAP126 | 0.956989247 | DAAM2 | 0.648888889 |

**Supplementary table 4.**  Top ten genes associated with death in the yellow module, and with intensive care in the magenta module. AUC = area under the receiver operating characteristic curve. The number of cases in the individual groups of death (n = 3) or need for intensive care (n = 9) are too low to make robust conclusions.
